# Pancreaticopleural fistula causing pleural effusion: a case report and review of the literature

**DOI:** 10.1186/s13256-024-04457-8

**Published:** 2024-03-29

**Authors:** Milan Khadka, Suzit Bhusal, Binod Pantha, Rabin Gautam, Kapil Gautam, Ashlesha Chaudhary

**Affiliations:** 1 Department of Medicine, National Academy of Medical Sciences, Kathmandu, 44600 Nepal; 2 National Trauma Center, Kathmandu, 44600 Nepal; 3 Everest Hospital Pvt Ltd, Kathmandu, Nepal

**Keywords:** Pancreaticopleural fistula, Acute necrotizing pancreatitis, Pleural effusion, Octreotide therapy, Case report

## Abstract

**Background:**

Pancreaticopleural fistula is a rare complication of pancreatitis and poses diagnostic and therapeutic challenges. This case report sheds light on the unique challenges posed by pancreaticopleural fistula as a rare complication of pancreatitis. The aim is to contribute valuable insights to the scientific literature by presenting a case involving a middle-aged man with acute necrotizing pancreatitis and associated pleural effusion.

**Case presentation:**

A 41-year-old Asian male with a history of pancreatitis and chronic alcohol use presented with severe dyspnea, chest pain, and left-sided pleural effusion. Elevated serum amylase lipase levels and imaging confirmed acute necrotizing pancreatitis with a computed tomography severity index of 8/10. Magnetic resonance cholangiopancreatography revealed pancreatic necrosis and pseudocyst formation and findings suggestive of pancreaticopleural fistula. The patient was then treated with octreotide therapy.

**Conclusion:**

The management of pancreaticopleural fistula demands a comprehensive and individualized approach. Recognition guided by high clinical suspicion coupled with appropriate investigations and a careful balance between medical, endoscopic, and surgical interventions is crucial for achieving favorable outcomes. This case report adds to the scientific literature by providing insights into the complexities of pancreaticopleural fistula and emphasizing the importance of personalized strategies in its management.

## Background

Pancreaticopleural fistula (PPF) is a rare complication associated with pancreatitis, particularly chronic pancreatitis, which presents a unique challenge in diagnosis and management [1]. Chronic pancreatitis, often linked to prolonged alcohol abuse, constitutes a significant etiological factor for PPF [2]. PPF arises when pancreatic secretions dissect through fascial planes, forming a communication into the retroperitoneum and subsequently into the pleural cavity [3, 4]. This case report focuses on the complex manifestations and therapeutic considerations in a 41 year old male with a history of alcohol-induced pancreatitis, showcasing the plausible relationship between pancreatic pathology and pleural involvement. This case report has been reported in accordance with the case report and literature review (CARE) checklist [5]

## Case presentation

A 41-year-old Asian male presented to the medicine department with a 2-month history of progressively worsening shortness of breath, exacerbated over the past 7 days. The dyspnea was insidious in onset, reaching Modified Medical Research Council Dyspnea Scale grade IV (MMRC IV), and worsened while sleeping on the right lateral side. Paroxysmal nocturnal dyspnea (PND) was observed, accompanied by chest pain. The patient developed left-sided chest pain, abdominal pain, and anxiety, with a blood pressure of 150/100 mmHg and SpO_2_ of 70%. The patient had a past history of multiple episodes of pancreatitis, which were managed supportively. He was diagnosed as having alcohol-induced pancreatitis, and he had a significant daily alcohol consumption for over 10 years. His family and psychosocial history were irrelevant.

Upon examination, reduced air entry was noted on the left side. After the initial assessment, the patient received intravenous fluids, analgesics, and oxygen therapy. The vitals were then stabilized, and the patient was admitted following the diagnosis of left-sided pleural effusion. Initial laboratory investigations revealed normal complete blood count (CBC), urine routine examination (RE), renal function test (RFT), liver function test (LFT), glucose, and coagulation profile.

**Fig. 1:**
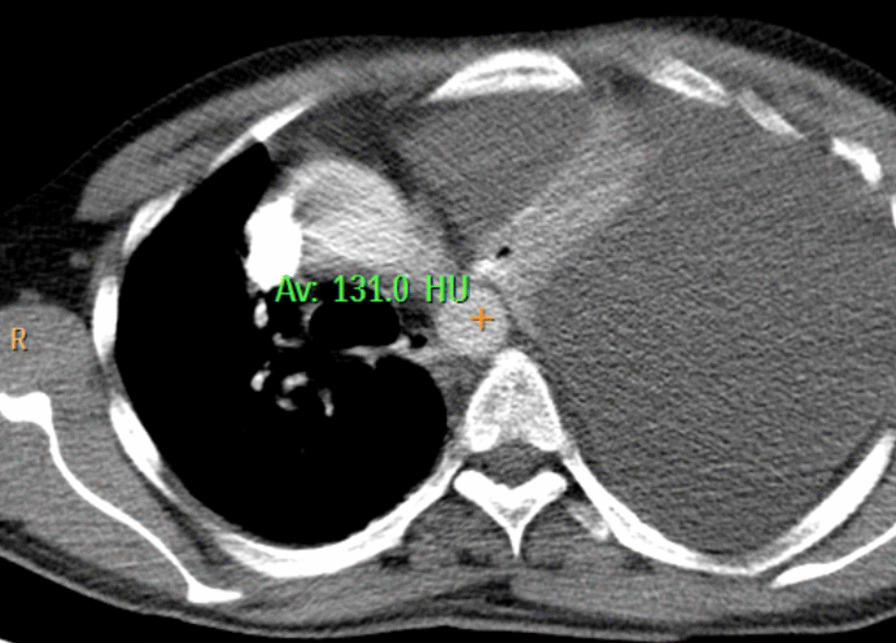
Computed tomography scan showing gross free fluid in the left pleural cavities

Ultrasonography demonstrated a significant left pleural effusion, later confirmed to be around 1 L. The computed tomography (CT) scan indicated pancreatic measurements of 25 mm, 13 mm, 16 mm, and 14 mm in the head, neck, body, and tail regions, respectively. Pancreatic inflammation, peripancreatic fat strandings, and minimal collections were observed. The parenchyma showed heterogeneous enhancement with non enhancing areas. Gross free fluid in the left pleural cavities, along with left lung collapse/consolidation, suggested acute necrotizing pancreatitis. The modified CT severity index was calculated as 8/10 (severe) on the basis of pancreatic inflammation, necrosis, and extrapancreatic complications (Fig. 1).

**Fig. 2:**
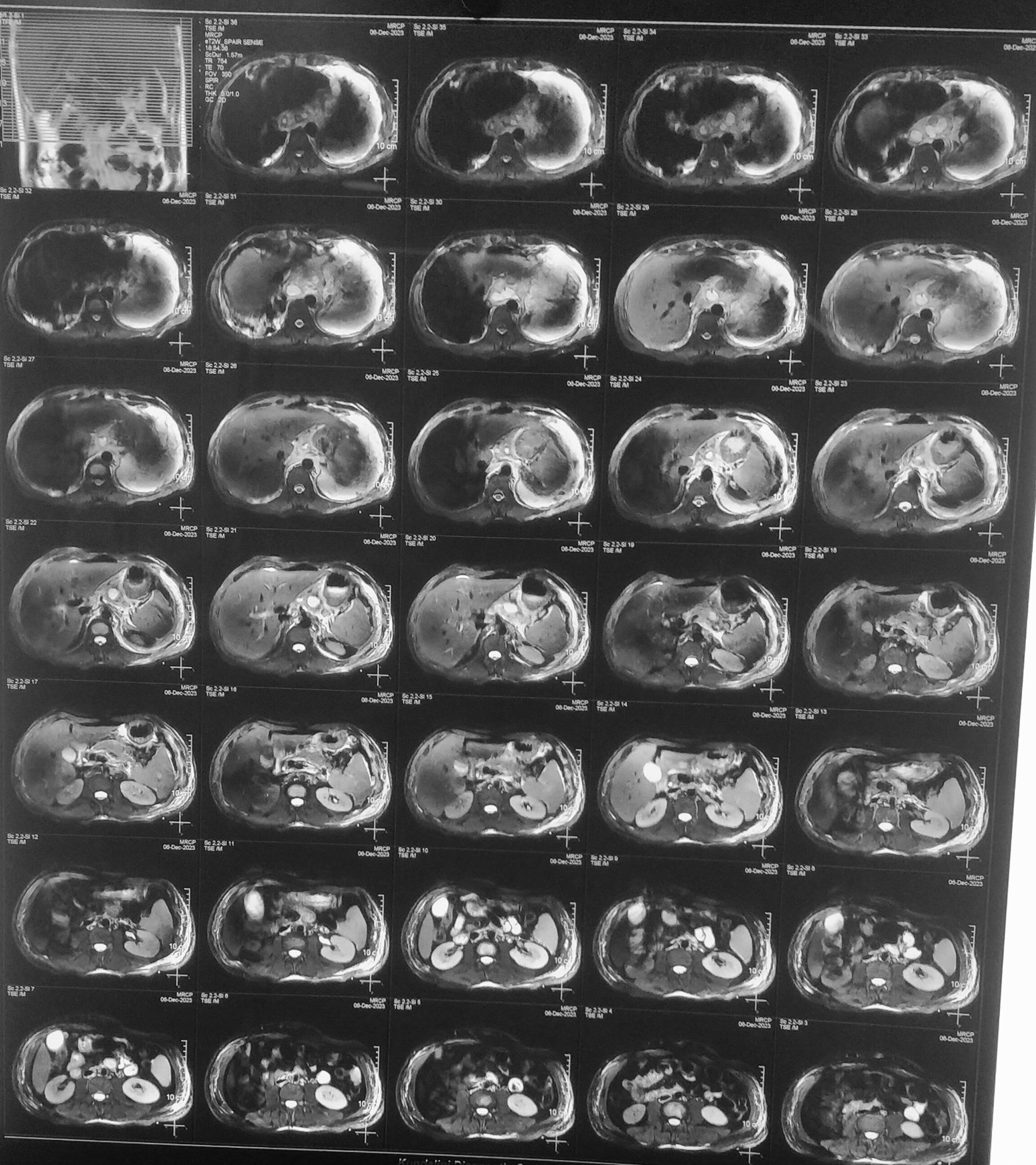
Magnetic resonance cholangiopancreatography images demonstrating heterogeneous signal of the pancreas, ill-defined margins, and fluid surrounding the pancreas

Magnetic resonance cholangiopancreatography (MRCP) revealed a heterogeneous signal of the pancreas, ill-defined margins, and fluid surrounding the pancreas suggestive of necrotic pancreatitis with potential pseudocyst formation and extension into the chest cavity (Fig. 2).

**Fig. 3:**
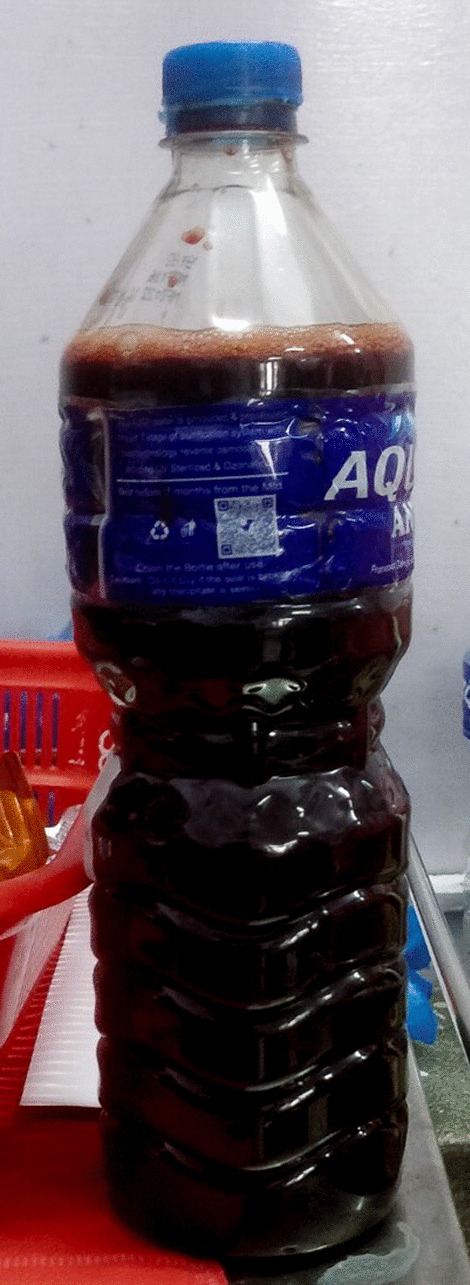
Dark-red pleural fluid from the patient’s left pleural cavity

Diagnostic and therapeutic aspiration of the pleural fluid was done, which revealed dark red hemorrhagic pleural fluid indicative of hemorrhage within the pleural cavity. Approximately 1000 ml of pleural fluid was drained revealing large volume of hemorrhagic effusion. The fluid, while not overtly turbid, displayed a subtle cloudiness likely attributed to the suspended red blood cells. No distinct or abnormal odor was noted during the examination, indicating the absence of infectious or purulent components within the hemorrhagic pleural fluid. The consistency of the fluid was noted to be watery, in line with the typical characteristics of pleural fluid. No unusual viscosity or thickening was observed. (Fig. 3). Following the aspiration, the patient had some symptomatic relief.

Pleural fluid analysis showed protein of 5.3 g/dl, glucose of 71 mg/dl, total leukocyte count (TLC) of 700/mg, with 20% neutrophils, 80% lymphocytes, and the presence of red blood cells (RBCs). Lactate dehydrogenase (LDH) levels in the pleural fluid increased from 910 U/L to 1738 U/L comparing the initial assessment of fluid and subsequent assessment, which may suggest increasing cellular damage within the pleural space. Serum amylase lipase levels were elevated at 224/514. Pleural fluid adenosine deaminase (ADA) was 33.8 µ/l, amylase 12442, and the cell count was 350 (N60 L50). These findings collectively contributed to the diagnosis of left-sided pleural effusion, acute necrotizing pancreatitis, and pancreaticopleural fistula.

An electrocardiogram (ECG) revealed sinus tachycardia and q-wave in I avL V5–V6. Following this, a definitive diagnosis of left-sided pleural effusion, acute necrotizing pancreatitis, and pancreaticopleural fistula was made, and the patient was started on octreotide therapy three times daily along with other supportive treatment and underwent regular observation and follow-up to monitor the progress of pancreaticopleural fistula secondary to acute necrotizing pancreatitis and associated pleural effusion with positive outcomes. The treatment approach involved a comprehensive and individualized strategy, considering medical, endoscopic, and surgical interventions as deemed appropriate.

The patient was closely monitored with regular clinical evaluations to assess symptoms, vital signs, and overall well-being. The patient’s response to octreotide therapy was carefully evaluated, focusing on reductions in fistula output and improvements in clinical symptoms. The patient’s symptomatic relief and improvements in quality of life were considered, emphasizing factors such as the resolution of dyspnea, chest pain, and overall respiratory distress. Upon achieving significant clinical improvement and stabilization of the pancreaticopleural fistula (PPF) and associated pleural effusion, the patient was considered for discharge. The patient and caregivers were educated on recognizing early signs of complications or worsening symptoms, emphasizing the importance of prompt medical attention if such issues arose. Dietary recommendations, especially related to alcohol consumption, were provided to promote overall health and prevent future exacerbations of pancreatitis. The patient received comprehensive education regarding the nature of pancreaticopleural fistula, the importance of medication adherence, and lifestyle modifications to prevent recurrence.

## Discussion

The presented case highlights the nature of pancreaticopleural fistula (PPF), a rare complication predominantly associated with chronic pancreatitis and alcohol abuse. The patient’s clinical course, marked by severe respiratory distress and left-sided pleural effusion shows the interplay between chronic alcohol-induced pancreatitis, pancreatic duct disruption, and subsequent pleural involvement, contributing to a refractory and rapidly accumulating pleural effusion. Clinical examination, laboratory investigations, and advanced imaging techniques collectively contributed to the formulation of an accurate diagnosis. Initiation of octreotide therapy was deemed appropriate based on the observed benefits in reducing fistula output and expediting closure. The decision to opt for initial medical management aligns with the evolving trend in PPF treatment, emphasizing a less invasive approach before considering alternative interventions. Regular observation and follow-up are integral components of the comprehensive management plan, allowing for dynamic adjustments based on the patient’s response.

Pancreaticopleural fistula (PPF) remains a rare but challenging complication associated with chronic pancreatitis. Recognition of PPF as a distinct clinical entity has evolved since the late 1960s [6]. The classic description often involves middle-aged, chronic alcoholic males presenting with breathlessness [7]. Males constitute the majority of cases, and PPF-mediated pleural effusion predominantly manifests with dyspnea [2, 8]. The presented case aligns with this profile, emphasizing the importance of clinical suspicion in such scenarios.

The diagnosis of PPF involves differentiating it from reactive effusions in acute pancreatitis, considering its refractory nature and rapid accumulation [1, 9, 10]. Elevated pleural fluid amylase levels, a characteristic finding, serve as a key diagnostic marker [11]. However, challenges arise due to various pathologies with increased amylase levels [12, 13]. Hence, a high index of suspicion in the right clinical context is crucial for accurate diagnosis. Imaging modalities play a pivotal role in delineating PPF and guiding therapeutic decisions. Computed tomography (CT) is valuable for detecting pleural effusion and associated pancreatic parenchymal changes [1, 10]. Magnetic resonance cholangiopancreatography (MRCP) offers noninvasive visualization of ductal anatomy, aiding in stratifying further management [14, 15],

While there is no established optimum therapy for PPF, initial conservative approaches involving medical therapy and observation are common [1, 10]. Somatostatin analogs, such as octreotide, play a crucial role in reducing fistula output and expediting closure. However, the duration of medical treatment remains a critical question, with a suggested period of 2–4 weeks before considering alternative interventions [16]

Endoscopic procedures, particularly endoscopic retrograde cholangiopancreatography (ERCP) with stent placement, have revolutionized nonoperative therapy for PPF [1, 12]. Stents serve dual roles in mechanically occluding the fistulous communication and dilating duct strictures [10]. Success rates vary, emphasizing the importance of patient selection based on ductal anatomy [17]. Surgical intervention, though definitive, is typically reserved for cases unresponsive to medical or endoscopic treatments [1, 10]. Exploration of the pertinent medical literature reveals the multifaceted landscape of PPF, emphasizing its association with chronic pancreatitis and its atypical manifestation in the form of pleural effusion. The diagnostic challenges are highlighted, with elevated pleural fluid amylase serving as a key marker. The evolving role of imaging modalities, including CT and MRCP, is discussed in guiding accurate diagnosis and therapeutic decisions.

In comparing the presented case with similar instances in the literature, commonalities and differences emerge. Similar cases often involve middle-aged males with a history of chronic pancreatitis, particularly associated with alcohol consumption. The predominant symptomatology is dyspnea, consistent with respiratory distress observed in the present case. Elevated pleural fluid amylase levels, a hallmark of PPF, are consistently reported in the literature [10–14]. Contrastingly, the heterogeneity in disease manifestation and therapeutic outcomes is notable. While some cases advocate for a predominantly medical approach using somatostatin analogs, others favor endoscopic interventions such as endoscopic retrograde cholangiopancreatography (ERCP) with stent placement. Surgical intervention, though definitive, is typically reserved for cases unresponsive to initial measures [11].

Hence, a comprehensive analysis of the patient’s presentation, diagnostic workup, and therapeutic response is crucial. The rationale behind favoring an initial medical approach with octreotide and regular observation lies in the observed benefits of somatostatin analogues in reducing fistula output and expediting closure. The consideration of endoscopic interventions, guided by imaging findings, aligns with the evolving trend in PPF management. Surgical intervention, while recognized as definitive, is reserved for cases unresponsive to initial measures, emphasizing a nuanced and stepwise approach tailored to the individual patient.

The strength of our case lies in the systematic utilization of imaging, laboratory data, and clinical observations to inform therapeutic decisions. The incorporation of evolving endoscopic techniques reflects a commitment to contemporary, less invasive interventions. However, limitations include the rarity of PPF, which affects the generalizability of findings, and the absence of a standardized treatment algorithm. Additionally, as this is a case report, it has inherent limitations in controlling variables and establishing causation.

## Conclusion

This case sheds light on the management of pancreaticopleural fistula (PPF), a rare complication often associated with chronic pancreatitis and alcohol abuse. The presented insights emphasize the importance of considering PPF in the differential diagnosis of pleural effusions, particularly in the context of a history of pancreatitis. The diagnostic significance of elevated pleural fluid amylase levels and the evolving role of advanced imaging techniques, notably MRCP, offer crucial guidance for accurate diagnosis. The case highlights the necessity for a tailored and stepwise approach, incorporating medical, endoscopic, and surgical interventions, as deemed appropriate, to optimize patient outcomes.

## Data Availability

All data generated or analyzed during this study are included in this article.

## Abbreviations

PPF: Pancreaticopleural fistula
CT: Computed tomography
MRCP: Magnetic resonance cholangiopancreatography
CARE: Case report and literature review
CBC: Complete blood count
Urine RE: Urine routine examination
RFT: Renal function test
LFT: Liver function test
ECG: Electrocardiogram
ADA: Adenosine deaminase
MMRC IV: Modified Medical Research Council Dyspnea Scale grade IV
PND: Paroxysmal nocturnal dyspnea
SpO2: Oxygen saturation
LDH: Lactate dehydrogenase
ERCP: Endoscopic retrograde cholangiopancreatography
